# Dynamic multi-omics mechanisms underpinning retinol tolerance: stage-specific reconstruction of skin barrier function and host–microbiome metabolic interactions

**DOI:** 10.3389/fmicb.2025.1668712

**Published:** 2025-10-27

**Authors:** Yixuan Huang, Qi Zhou, Minyan Gui, Danni Guo, Jingmin Cheng, Wentao Ma, Peng Shu, Xiao Liu

**Affiliations:** ^1^Tsinghua Shenzhen International Graduate School, Tsinghua University, Shenzhen, Guangdong, China; ^2^HBN Research Institute and Biological Laboratory, Shenzhen Hujia Technology Co., Ltd., Shenzhen, Guangdong, China; ^3^Guangdong Engineering Technology Research Center for Functional Skincare Innovation, Shenzhen Hujia Technology Co., Ltd., Shenzhen, Guangdong, China

**Keywords:** skin barrier function, host-microbiome metabolic interactions, retinol tolerance, skin microbiome, multi-omics, metabolomics, systems microbiology

## Abstract

**Background:**

Retinol remains an essential component in anti-aging skincare; however, a subset of users develop intolerance, characterized by compromised barrier integrity and inflammation. The temporal dynamics of how skin microbiota and host metabolism co-evolve during retinol tolerance establishment remain poorly understood.

**Methods:**

We conducted a prospective 28-day longitudinal study with 18 Chinese women (aged 25–40): 9 retinol-intolerant subjects monitored at baseline, adverse reaction phase, and tolerance establishment, while baseline data from 9 retinol-tolerant individuals served as controls. We integrated cutaneous phenotypic measurements, metagenomic sequencing, and untargeted metabolomics.

**Results:**

In the intolerant group, skin phenotype assessment revealed a distinct biphasic response—an acute phase marked by increased stratum corneum hydration, reduced sebum secretion, lower skin pH, and improved wrinkle metrics, followed by a re-equilibration phase characterized by sustained barrier restoration. Metagenomic profiling of 969 microbial species demonstrated that, although overall microbial α-diversity remained stable across time points in both groups, key taxa in the intolerant group exhibited transient “rise-and-fall” dynamics. At baseline, the intolerant group exhibited overrepresentation of Cutibacterium acnes, whereas the tolerant group was enriched in potentially protective species, including *Sphingomonas hankookensis* and *Acinetobacter johnsonii*. Untargeted metabolomics showed marked temporal fluctuations with an initial phase of metabolic turbulence, followed by partial recovery. During the early adverse reaction phase in intolerant subjects, lipid and fatty acid metabolic pathways—specifically, glycerophospholipid, linoleic acid, α-linolenic acid, and ether lipid metabolism—were significantly upregulated, concomitant with the suppression of TCA cycle and sphingolipid activity. Conversely, as tolerance was established, enhanced activity in the TCA cycle, sphingolipid, ascorbate, and pentose metabolism pathways—coupled with a reduction in pro-inflammatory arachidonic acid derivatives-indicated metabolic reconstitution and restoration of barrier integrity.

**Discussion:**

Integrated multi-omics correlation analyses further underscored the tightly interconnected regulation of host-microbe energy metabolism, antioxidant defenses, and membrane repair in response to retinol-induced stress. These findings elucidate the temporal interplay between host and microbial processes underpinning retinol tolerance and highlight baseline biomarkers that may facilitate personalized skincare interventions.

## 1 Introduction

Retinol (vitamin A alcohol), an active derivative of vitamin A and a precursor to retinoic acid, promotes epidermal keratinocyte differentiation, modulates sebaceous gland function, inhibits extracellular matrix degradation, and stimulates collagen synthesis (Fisher and Voorhees, 1998; Kang et al., 1995; Berenguer and Duester, 2022). Compared to retinoic acid—a potent skin irritant—retinol exhibits milder effects and provides superior cutaneous tolerance, rendering it widely applicable for the treatment and prevention of photoaging, acne, hyperpigmentation, and wrinkles (Kong et al., 2016; Kikuchi et al., 2009; Mukherjee et al., 2006; Tucker-Samaras et al., 2009; Farris, 2022).

Owing to its lipophilic properties, retinol readily penetrates skin cells and undergoes a two-step metabolic conversion into its bioactive form. Initially, alcohol dehydrogenases (ADHs) oxidize retinol to retinaldehyde, which is subsequently converted to retinoic acid by retinaldehyde dehydrogenases (RALDH1, RALDH2, and RALDH3) (Szymański et al., 2020; Ghyselinck and Duester, 2019; Roos et al., 1998; Napoli and Yoo, 2020). Retinoic acid regulates gene expression by binding to nuclear retinoic acid receptors (RARs) and retinoid X receptors (RXRs) (Kurlandsky et al., 1994; Kafi et al., 2007; Cosio et al., 2021). The anti-aging effects of retinol have been extensively studied, and evidence shows that it can induce epidermal thickening, renew the stratum corneum via the activation of epidermal stem cells, and promote keratinocyte proliferation and differentiation (Kang et al., 1995; Duell et al., 1996; Becker et al., 2020). Additionally, retinol enhances the expression of collagen, fibronectin, and elastin in dermal fibroblasts, thereby improving skin elasticity and reducing wrinkle formation (Quan, 2023; Shao et al., 2017).

Despite these benefits, clinical studies indicate that a considerable proportion of first-time users experience adverse reactions–such as erythema, burning, stinging, and desquamation–that can compromise treatment adherence and limit clinical utility (Zasada and Budzisz, 2019; Levin and Momin, 2010). Here, we use the term “retinol intolerance” operationally to denote the well-documented, transient retinoid-induced irritation—often referred to in the dermatology literature as ‘retinoid dermatitis' or ‘retinoid reaction'—characterized by erythema, burning/stinging, and desquamation (Levin and Momin, 2010; Zasada and Budzisz, 2019; Fluhr et al., 1999; Dispenza et al., 2012; Kim et al., 2003). This usage distinguishes these irritant reactions from true allergic contact dermatitis, which is immunologically mediated and less common. Traditionally, retinol-induced skin intolerance has been ascribed to impaired stratum corneum barrier function, elevated release of inflammatory cytokines (e.g., IL-1α and IL-8), and excessive activation of Toll-like receptors (Kim et al., 2003; Fluhr et al., 1999; Dispenza et al., 2012). Notably, most users eventually develop tolerance after several weeks of continuous use (Culp et al., 2015), which suggests the presence of adaptive mechanisms. Existing research has primarily focused on single-dimensional phenotypic observations or on the identification of specific molecular biomarkers, leaving the comprehensive biological mechanisms underlying tolerance establishment poorly understood (Fang et al., 2024; Ma et al., 2022). However, the temporal dynamics of how skin microbiota and host metabolism co-evolve during retinol tolerance establishment remain poorly understood, limiting our ability to predict and mitigate adverse reactions.

As the body's largest organ, the skin harbors millions of commensal microorganisms, collectively known as the skin microbiome (Byrd et al., 2018). These microbial communities play pivotal roles in pathogen resistance, immune modulation, and barrier reinforcement (Harris-Tryon and Grice, 2022). A growing body of evidence demonstrates that skin ecosystems are highly responsive to exogenous stimuli, with alterations in community structure and function facilitating host adaptation (Kong et al., 2012; Williams and Gallo, 2015). In addition, retinol exhibits antimicrobial properties that further enhance cutaneous defense (Pechere et al., 2002; Park et al., 2023). For instance, Harris et al. (2019) reported that dietary vitamin A stimulates epidermal production of the antimicrobial protein RELM, implying a protective role against pathogenic bacterial colonization. Moreover, vitamin A deficiency has been linked to *Staphylococcus aureus* skin infections (Wiedermann et al., 1996; Roche and Harris-Tryon, 2021). Nevertheless, the impact of topical retinol on skin microbiota remains understudied, particularly regarding longitudinal monitoring and the integration of multi-omics analyses to decipher host-microbe interaction networks during tolerance development (Dreno et al., 2017).

In this study, we employed a prospective 28-day longitudinal design to investigate retinol tolerance mechanisms. We recruited 18 Chinese women aged 25–40 years with no recent retinol use, applying 0.25% retinol lotion daily. Participants were categorized as retinol-tolerant (*n* = 9, no adverse reactions) or retinol-intolerant (*n* = 9, initial reactions but tolerance by day 28). We integrated cutaneous phenotypic measurements, metagenomic sequencing, and untargeted metabolomics to address: (1) skin phenotype dynamics during tolerance establishment, (2) microbial community remodeling in response to retinol, and (3) host-microbe metabolic interactions in tolerance development. We hypothesized that tolerance involves coordinated changes in barrier function, microbial dynamics, and metabolic interactions, with baseline biomarkers differentiating tolerant from intolerant individuals.

## 2 Materials and methods

### 2.1 Study design and subject recruitment

This study enrolled eighteen Chinese women, aged 25–40, who had not used any retinol products in the month preceding enrollment (Trial Registration Number: ChiCTR2400089010). Among these participants, nine were categorized as the retinol-intolerant group, as they met the criteria of experiencing adverse reactions within the first week of initial application but achieving tolerance by day 21 or 28. For comparison, data from nine retinol-tolerant participants were obtained from our previous study (Gui et al., 2024) using identical protocols. The other nine were classified as the retinol-intolerant group, as they met the criteria of experiencing adverse reactions within the first week of initial application but achieving tolerance by day 28. All participants agreed to abstain from using cosmetics, medications, or supplements that could influence the results. They maintained their regular lifestyles throughout the study and provided written informed consent after receiving a detailed explanation of the protocol. Prior to enrollment, a dermatologist assessed each participant's skin condition and conducted a safety evaluation following the Technical Code of Cosmetic Safety (2015 edition, China).

Skin reactions were evaluated by a board-certified dermatologist using a standardized 5-grade classification system (0 = no reaction, 1 = mild erythema, 2 = erythema with infiltration and papules, 3 = erythema with edema, papules and vesicles, 4 = erythema with edema and bullae). Adverse reactions were defined as Grade 1 or higher responses.

Subjects were included if they met all of the following criteria: (1) Healthy female subjects, aged between 25 and 40 years; (2) Subjects presenting with a dull and lackluster skin complexion; (3) Ability to cooperate fully with study procedures and maintain a regular lifestyle throughout the study period; (4) Capacity to read, comprehend, and voluntarily sign the informed consent form; (5) Agreement to refrain from using any cosmetics, medications, or health supplements that might affect the study outcomes during the trial; (6) Given that the primary objective of this study is to investigate changes in skin micro ecology during the development of retinol intolerance, subjects were categorized into two groups: the retinol-intolerant group (*n* = 9), comprising individuals who experienced adverse reactions within one week of initiating retinol use but subsequently developed complete tolerance within 28 days, and the retinol-tolerant group (*n* = 9), comprising subjects who remained continuously tolerant throughout the study period; (7) Any other relevant inclusion criteria as applicable.

Subjects were excluded if they met any of the following criteria: (1) Presence of facial skin diseases likely to influence test outcomes; (2) high sensitivity or severe allergies; (3) pregnancy, breastfeeding, or plans to become pregnant during the trial; (4) severe cardiac, hepatic, or renal impairments, or marked immunosuppression; (5) psychiatric or severe endocrine disorders, or current use of oral contraceptives; (6) participation in any drug clinical trial or other experimental study within the past 30 days or recent systematic use of medications that might confound test results; (7) use of any cosmetic products–either topically or orally–within the previous two weeks; (8) inability to comply with the study procedures; (9) determination by the investigator that the subject was unsuitable for participation; (10) occurrence of adverse reactions during the trial that rendered continued participation unsafe, as judged by a dermatologist; or (11) any other condition deemed appropriate by the investigators to exclude a potential participant.

A skin lotion containing 0.25% retinol was used in this study. Participants applied the lotion once daily in the evening to one randomly assigned half of their face beginning on Day 0, while the other half remained untreated as an internal control. The dosage was verified by weighing the product at each follow-up visit. Skin swabs and facial phenotype data were collected from the treated side at three distinct time points: Day 0 (baseline), Day 7 (when adverse reactions occurred), and either Day 21 or Day 28 (when tolerance was established).

### 2.2 Sample collection

Samples were collected weekly under strictly controlled conditions, maintaining a regulated temperature of 21±1°C and humidity of 50 ± 10% RH. On each sampling day, participants maintained their natural facial microenvironment and refrained from washing their faces for at least 12 h prior to collection.

Two adjacent 2 cm × 2 cm areas on the cheeks were designated as the “DNA sampling area” and the “metabolite sampling area.” Sterile cotton-tipped swabs (Winner, China) and a 0.9% NaCl saline solution were used to collect microbial DNA and metabolite samples, respectively. The collected swabs were immediately stored at –30°C until further processing. After sample collection, participants cleansed their faces, dried their skin, and rested for 30 min in a controlled laboratory environment before phenotype measurements were taken.

Facial phenotype data were collected using the following instruments: the Corneometer CM 825 (Courage + Khazaka Electronic GmbH, Cologne, Germany) for stratum corneum water content (WCSC), the Vapometer for transepidermal water loss (TEWL), the Skin-pH-Meter pH905 for skin surface pH, and the Sebumeter SM815 (Courage + Khazaka Electronic GmbH, Cologne, Germany) for sebum content. Additionally, facial imaging was performed with the VISIA-CR system (Canfield Scientific, USA) to assess porphyrin area, pore area, the percentage of skin red zone, and skin color values (L*, a*, and b*). Wrinkle indicators at the corners of the eyes–including wrinkle number, length, area, and volume–were evaluated using the optical imaging system PRIMOS-CR (Canfield Scientific, USA).

Participant recruitment, the management of subjects, skin microbiome and metabolome sampling, as well as phenotype measurements were conducted by Weipu Testing Technology Group Co., LTD (China) under a contract research arrangement. All participants provided written informed consent after fully understanding the study procedures.

### 2.3 Microbiome DNA extraction and library preparation

Genomic DNA from skin microbiome swab samples was extracted using the DNeasy PowerSoil Pro Kit (Qiagen) according to the manufacturer's protocol. Briefly, the swab samples were lysed in lysis buffer and vortexed with zirconium beads for 15 min. The resulting crude lysate was then subjected to inhibitor removal for purification. The purified lysate was mixed with a DNA binding solution and passed through a silica spin filter membrane. Following a two-step washing process, the silica-bound DNA was eluted with 100 μL of 10 mM Tris elution buffer. DNA concentration was quantified using a Qubit fluorometer (Thermo Fisher Scientific), and the extracted samples were stored at −30°C until library preparation.

Metagenomic DNA libraries were prepared using the VAHTS Universal Plus DNA Library Prep Kit for MGI (Vazyme), following the manufacturer's instructions. Single-indexed paired-end libraries with an average insert size of 150 bp were generated for each sample. DNA fragmentation, adapter ligation, and library amplification were performed according to the kit protocol, with 17 cycles of PCR. The libraries were purified using VAHTS DNA Clean Beads (Vazyme) and sequenced on the DNBSEQ-T7 platform (MGI Tech, China) by Geneplus.

### 2.4 Metagenomics sequencing data analysis

Metagenomic data were processed using the ATLAS pipeline (Kieser et al., 2020) (v2.16.3) for quality control, assembly, genomic binning, and annotation. Briefly, PCR duplicates were removed using BBTools, and reads were quality-trimmed, with contaminating sequences from PhiX and the human genome filtered out. Clean reads were then error-corrected and merged prior to assembly using MEGAHIT (Li et al., 2015). Assembled contigs were further quality-filtered, and their coverage was estimated using BBMap.

On average, each sample yielded 44.6 ± 18.3M raw reads. After quality control, host-derived reads accounted for approximately 75.6% of the total, while microbial reads constituted 24.4%. Overall, the 27 samples yielded a total of 292.45M clean reads, with an average of 10.8 ± 8.6M reads per sample. Taxonomic classification of clean reads was performed using mOTUs3 (Ruscheweyh et al., 2021) (v3.0.3 on Python v3.8.12), which clustered reads into operational taxonomic units (OTUs) based on 97% sequence similarity and annotated them with relevant reference databases. Additionally, HUMAnN3 (Beghini et al., 2021) (v3.6) was used to quantify gene families, with functional definitions provided by the UniRef and KEGG databases. Pathway enrichment analysis was conducted using the ReporterScore metric (Peng et al., 2023), with significantly enriched pathways defined as those having an absolute score of at least 1.96 (i.e., —ReporterScore— 1.96).

Two sequential comparisons were conducted: (1) comparing the intolerance state (when adverse reactions occurred) to the baseline (Day 0); and (2) comparing the established tolerance state to the intolerance state. In each comparison, the sign of the ReporterScore indicates the direction of regulation, with positive values denoting upregulation and negative values indicating downregulation relative to the control condition for that stage.

### 2.5 LC-MS metabolomic data collection and analyses

Non-targeted metabolomic experiments and data preprocessing were conducted by Shanghai OE Biotech Co., Ltd. (China). Briefly, two skin swab samples were collected from each participant into a 5 mL centrifuge tube and mixed with 1 mL of pre-cooled methanol-water (4:1, v/v; Thermo Fisher). The samples were sonicated briefly in an ice-water bath for 20 min and then stored at −40°C overnight. Subsequently, the samples were centrifuged for 10 min at 12,000 rpm at 4°C, and 500 μL of the supernatant was transferred into an LC-MS injection vial.

Next, the samples were re-dissolved in 300 μL of methanol-water (1:4, v/v) containing a mixed internal standard at a concentration of 4 μg/mL. The mixed internal standard contained L-2-chlorophenylalanine, succinic acid-d4, L-valine-d8, and cholic acid-D4 to ensure accurate quantification across different metabolite classes. After vortexing for 30 s and sonicating for 3 min in an ice-water bath, the samples were stored at −40°C for 2 h. The samples were centrifuged again for 10 min at 12,000 rpm 4°C. Subsequently, 150 μL of the supernatant was aspirated using a syringe, filtered through a 0.22 μm organic-phase pinhole filter, transferred to a new LC injection vial, and stored at −80°C until LC-MS analysis.

For quality control (QC) sample preparation, equal volumes of extracts from all samples were combined and pooled. The extracted skin swab samples were analyzed using a Waters ACQUITY UPLC I-Class Plus system coupled with a Thermo QE Plus mass spectrometer, equipped with an ACQUITY UPLC HSS T3 column (100 mm × 2.1 mm, 1.8 μm).

Prior to pattern recognition, raw data were preprocessed using Progenesis QI v3.0 software (Nonlinear Dynamics, Newcastle, UK) for baseline filtering, peak identification, integration, retention time correction, peak alignment, and normalization. Metabolites were identified based on multiple criteria, including retention time (RT), accurate molecular weight, secondary fragmentation patterns, and isotopic distribution, with annotations referenced against the Human Metabolome Database (HMDB), Lipidmaps (v2.3), METLIN, and an in-house LuMet-Animal database. The resulting data matrix was refined by removing peaks with missing values in more than 50% of the samples. Remaining missing values were imputed with half of the minimum observed value.

Compound identification was evaluated using an 80-point scoring system based on the following criteria:

- Exact molecular weight match of the primary mass spectrum (20 points)
- Fragmentation match of the secondary mass spectrum (20 points)
- Isotopic distribution match (20 points)
- Retention time match (20 points)

Compounds with a total score of 36 or lower were considered inaccurately characterized and were excluded from further analysis. Lipid annotations followed the guidelines of the International Lipid Classification and Nomenclature Committee (ILCNC). Identification confidence levels were assigned based on available analytical evidence: Level 1 (authentic standard match), Level 2 (MS/MS spectral library match), Level 3 (tentative candidates based on fragmentation patterns), and Level 4 (unequivocal molecular formula). Structural details such as specific double bond positions and stereochemistry were only reported for Level 1–2 identifications supported by authentic standards or comprehensive MS/MS fragmentation evidence. For Level 3–4 identifications, conservative annotations reflecting the composition level were used.

Additionally, metabolic pathway analysis was conducted using the web-based tool MetOrigin (Yu et al., 2022)^1^ to identify the origins of metabolites (host, bacterial, or both). MetOrigin integrates seven metabolite databases (KEGG, HMDB, BIGG, ChEBI, FoodDB, DrugBank, and T3DB) containing 314,915 non-redundant metabolites, of which 191,031 contain specific source information that can be classified into host (mammals), microbiota (archaea, fungi, bacteria), cometabolism (shared by both host and microbiota), food, drug, and environment categories. This origin-based classification enables more precise metabolic pathway enrichment analysis (MPEA) by performing separate pathway analysis for metabolites from different origins, rather than treating all metabolites as a single pool, thereby improving the accuracy of identifying organism-specific metabolic functions.

### 2.6 Statistical analysis and data visualization

Statistical analyses and data visualizations were performed using Microsoft Excel (Microsoft Inc., Redmond, WA, USA) and R software (RStudio v4.3.2, R Foundation for Statistical Computing, Vienna, Austria), employing the ggplot2 package (version 3.4.4). Differential abundance analyses of bacterial taxa, genes, metabolites, and alpha diversity indices across various time points and baseline measurements were performed using the Wilcoxon rank-sum test. When all three time points were available for a subject, paired Wilcoxon signed-rank tests were conducted to account for within-subject variability; otherwise, an unpaired Wilcoxon rank-sum test was applied, with this distinction noted in the figure legends. Beta diversity was assessed using Bray-Curtis distances, and differences across time points and the baseline were evaluated with the Adonis test.

Spearman's correlation analyses were employed to investigate relationships among skin microbiota, metabolites, microbial genes, and skin phenotypes. Mantel tests were used to assess correlations between multivariate datasets. *P*-values were adjusted using the Benjamini & Hochberg (BH) method, with statistical significance defined as *p* < 0.05, unless otherwise specified.

## 3 Results

### 3.1 Phenotypic characterization of retinol-tolerant and -intolerant groups

**Figure 1:**
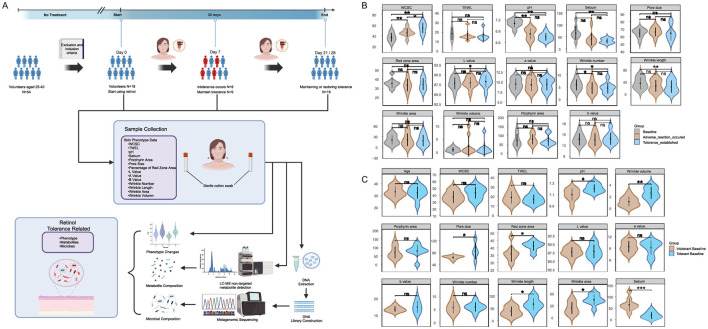
Study workflow and cutaneous phenotypic analyses: **(A)** Study design flowchart showing participant recruitment, intervention protocol, and classification timeline. The retinol-intolerant cohort (*N* = 9, current study) was identified based on adverse reactions at Day 7, with tolerance establishment monitored through Day 21–28. Data from the retinol-tolerant cohort (*N* = 9) were obtained from our previous study (Gui et al., 2024) for comparative analysis; **(B)** illustrates temporal changes in skin phenotypes within the retinol-intolerant group (*N* = 9) comparing Day 0, Day 7, Day 21/28 using paired analysis; and **(C)** compares baseline characteristics between the retinol-tolerant cohort (*N* = 9) from a previous study (Gui et al., 2024) and the current retinol-intolerant cohort (*N* = 9) using between-group analysis. Skin phenotype parameters include: WCSC, water content of the stratum corneum; TEWL, transepidermal water loss; pH, skin surface acidity; Sebum, sebum secretion level; Porphyrin area, porphyrin fluorescence area; Pore size, facial pore diameter; Percentage of red zone area, erythema coverage; Lab^*^ values, lightness, red-green, and yellow-blue chromaticity, with wrinkle metrics encompassing number, length, area, and volume. Statistical significance was determined using Wilcoxon matched-pairs tests with Benjamini-Hochberg (BH) correction, where *ns* designates non-significance, ^*^ denotes adjusted *p* ≤ 0.05, ^**^ adjusted *p* ≤ 0.01, and ^***^ adjusted *p* ≤ 0.001. The figure created with BioRender.com, accessed on 12 August 2025.

Following our comprehensive study protocol (Figure 1A), a longitudinal assessment of multidimensional skin phenotypes revealed dynamic biological responses to retinol intervention in the intolerant group (Figure 1B). During the acute phase (D0–D7), the skin barrier in the intolerant group rapidly adjusted following the daily application of the retinol moisturizing lotion. This adaptation was evidenced by a 23.5% increase in WCSC, a decrease of 0.28 units in skin surface pH, a 22.6% reduction in wrinkle count (likely reflecting optical smoothing rather than structural remodeling), and a 38.0% decline in sebum secretion compared to baseline. These early changes suggest retinol's potential regulatory influence on the epidermal microenvironment during the initial phase of application, though causality cannot be definitively established without placebo controls. By the tolerance establishment phase (D21/28), the skin in the intolerant group had achieved a state of homeostatic regulation. Hydration capacity remained elevated, with WCSC showing a 46.1% increase relative to baseline, while sebum secretion decreased by 49.6% compared to baseline. Additionally, wrinkle length decreased by 29.8% compared to baseline. Although hydration parameters continued to improve from D7 to D21/28 (Δ = +18.4%, *P* = 0.020), the other metrics stabilized (all *P*>0.05), indicating that functional barrier equilibrium was gradually achieved through phased regulation.

To identify baseline characteristics that may predispose individuals to different tolerance responses, we compared Day 0 measurements between the retinol-tolerant cohort from our previous study and the retinol-intolerant cohort from the current investigation (Figure 1C). The tolerant group exhibited unique microenvironmental characteristics, including a skin surface pH that was 0.11 units higher (*p* = 0.0336) and 70.6% lower sebum secretion (*p* = 0.0003) compared to the intolerant group. Additionally, structural evaluations showed a 32.4% larger pore area (*p* = 0.3865) and 14.4% greater erythema coverage (*p* = 0.0187) in the tolerant group. Notably, aging phenotypes were more pronounced in the tolerant group, which exhibited a 122.2% increase in wrinkle volume (*p* = 0.0040), along with significant increases in wrinkle length (*p* = 0.0151) and wrinkle area (*p* = 0.0142). After applying the Benjamini-Hochberg correction, the difference in sebum levels remained statistically significant (*adj*.*p* = 0.0043), whereas the difference in wrinkle volume was marginally significant (*adj*.*p* = 0.056).

These baseline differences represent inherent skin characteristics present before retinol exposure, suggesting their potential value as predictive biomarkers for retinol tolerance in clinical practice. Overall, these results demonstrate that baseline sebum secretion and wrinkle parameters could serve as potential biomarkers for predicting retinol tolerance, with the observed phenotypic changes prompting investigation of the underlying microbial mechanisms.

### 3.2 Dynamic characteristics of skin microbiota during retinol tolerance establishment

**Figure 2:**
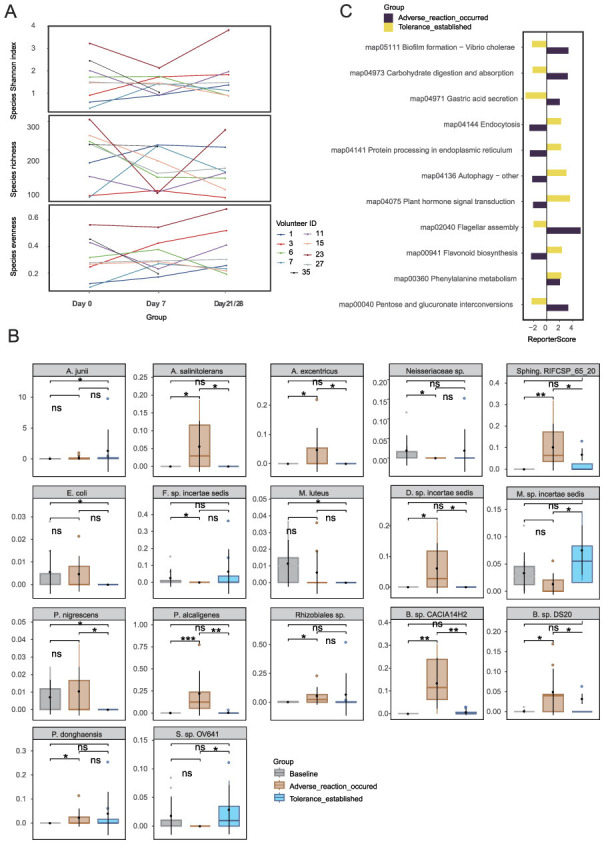
Microbial community dynamics during retinol tolerance establishment: **(A)** Species α-diversity analysis, **(B)** Stage-specific differential species, and **(C)** KEGG pathway analysis. Species abbreviations: *A. junii, Acinetobacter junii*; *A. salinitolerans, Agrobacterium salinitolerans*; *A. excentricus, Asticcacaulis excentricus*; *B. sp. CACIA14H2, Blastomonas sp. CACIA14H2*; *E. coli, Escherichia coli*; *F. sp. incertae sedis, Finegoldia sp. incertae sedis*; *M. luteus, Micrococcus luteus*; *M. sp. incertae sedis, Moraxella sp. incertae sedis*; *P. nigrescens, Prevotella nigrescens*; *P. alcaligenes, Pseudomonas alcaligenes*; *Rhizobiales sp., Rhizobiales* species; *Sphing. RIFCSP_65_20, Sphingomonadales bacterium RIFCSPHIGHO2_01_FULL_65_20*; *P. donghaensis, Porphyrobacter donghaensis*; *S. sp. OV641, Sphingomonas sp. OV641*; *B. sp. DS20, Brevundimonas sp. DS20*; *D. sp. incertae sedis, Dietzia sp. incertae sedis*; *Neisseriaceae sp., Neisseriaceae* species. *p < 0.05, **p < 0.01, ***p < 0.001.

Analysis using mOTUs3 and marker gene databases identified a total of 969 microbial species. The α-diversity analysis, performed using Wilcoxon matched-pairs tests with FDR correction, revealed no significant differences in evenness, richness, or the Shannon index across the baseline, intolerance, and tolerance phases (adj. *p*>0.05). Although species richness decreased during the intolerance phase, this reduction was not statistically significant (Supplementary Figure 1). Line chart (Figure 2A) further confirmed that the overall structure of the microbial community remained stable, maintained by functional redundancy within the community. This finding suggests that conventional α-diversity metrics may be insufficient to capture the subtle shifts associated with retinol tolerance.

Wilcoxon unpaired tests identified 17 species with stage-specific differential abundance (Figure 2B), including *Acinetobacter junii* (ref_mOTU_v3_00265), a resilient Gram-negative coccobacillus widely distributed in both clinical and natural environments (Bergogne-Berezin and Towner, 1996), which ranked among the top 20 most abundant taxa. In the intolerant cohort 17 species were differentially abundant over time. For comparison, 13 of these species were also detectable–but largely stable–in the tolerant group (Supplementary Figure 2). Seven species—including *Agrobacterium salinitolerans, Asticcacaulis excentricus, Blastomonas sp. CACIA14H2, Brevundimonas sp. DS20, Dietzia sp. incertae sedis, Pseudomonas alcaligenes*, and *Sphingomonadales bacterium*—exhibited a transient upregulation followed by a decline, implying their potential roles in early stress responses and subsequent repair processes. Notably, in the retinol-tolerant group, *Prevotella nigrescens* and *Sphingomonas sp*. exhibited significant changes between day 0 and day 21/28, may suggesting their involvement in the dynamic processes of retinol tolerance. Additionally, KEGG pathway analysis (|reporter score|≥1.96) identified three distinct regulatory patterns (Figure 2C). Early-activated pathways–such as pantothenate/CoA biosynthesis (map00040), BCG infection (map04973), and flagellar assembly (map02040)–suggest enhanced energy metabolism and motility during the initial adaptive phase. In later stages of the response, pathways such as flavonoid biosynthesis (map00941), map04075 (annotated as plant-pathogen interaction but representing conserved stress-response modules), and autophagy (map04136/04141) were enriched, indicating the induction of antioxidant defense mechanisms. Meanwhile, the sustained upregulation of phenylalanine metabolism (map00360) may contribute to skin homeostasis through its anti-inflammatory effects. Together, these findings demonstrate that while the overall structural composition of the skin microbiota remains stable during retinol tolerance development, a marked functional plasticity is evident through the dynamic regulation of specific species and metabolic pathways. The functional plasticity observed in microbial communities led us to examine metabolomic changes.

### 3.3 Dynamic changes of metabolites during retinol tolerance establishment

Untargeted metabolomics identified a total of 4,457 metabolites from skin swabs (Supplementary Table 1), including lipids, benzoids, and lignans. Among the 500 most abundant metabolites from the intolerant group, Wilcoxon matched-pairs tests with Benjamini–Hochberg correction (Supplementary Table 2) revealed that 409 metabolites (81.8% of the subset) exhibited significant abundance changes (*p* < 0.05) across the baseline (Day 0), adverse reaction (Day 7), and tolerance-established phases (Day 21/28). Notably, the most dramatic differences occurred during the transition from baseline to the adverse reaction phase (Day 0 → Day 7: 372 differential metabolites), followed by the adverse-to-tolerance transition (Day 7 → Day 21/28: 331 differential metabolites). Fewer changes were observed directly between baseline and tolerance phases (Day 0 → Day 21/28: 177 differential metabolites), indicating that metabolic alterations were most pronounced during the early phase, with a trend toward stabilization at the tolerance stage.

**Figure 3:**
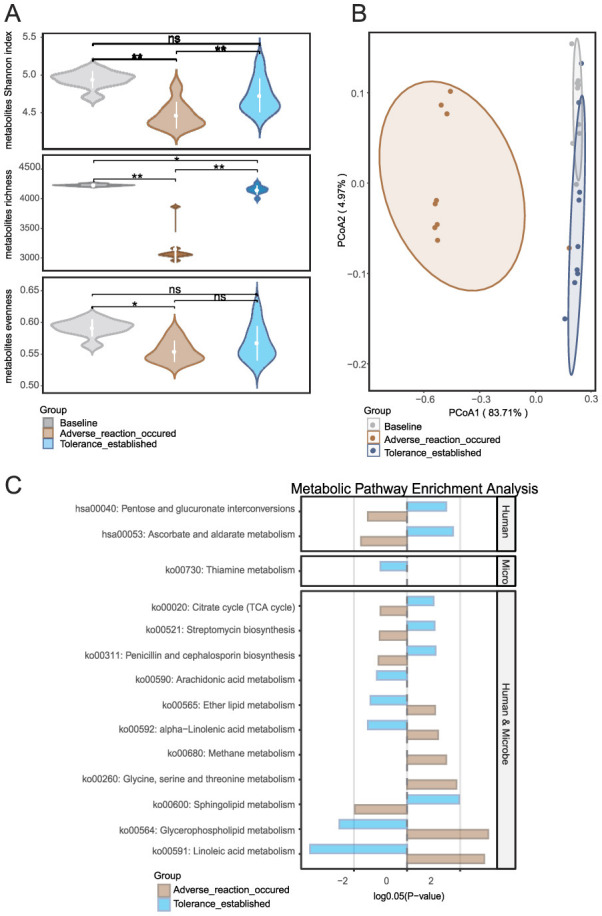
Metabolic landscape dynamics during retinol tolerance establishment: **(A)** Temporal changes in metabolite α-diversity, **(B)** Global evolution of metabolite composition as revealed by principal coordinate analysis (PCoA), and **(C)** Differential metabolite enrichment and origin analysis using MetOrigin software, which elucidates host–microbe metabolic interactions by comparing enrichment profiles across human, microbial, and co-sourced metabolites. Group comparisons for metabolite enrichment are shown between the baseline and intolerance phases, as well as between the intolerance and tolerance establishment phases. *p < 0.05, **p < 0.01.

Metabolite α-diversity analysis (Figure 3A) confirmed significant fluctuations between phases (*p* < 0.05), highlighting the high sensitivity of the metabolome to retinol stimulation. Principal coordinate analysis (PCoA) (Figure 3B) based on Bray-Curtis distances, combined with PERMANOVA (adonis, permutations = 9,999), further demonstrated precise spatiotemporal dynamics. The transition from baseline to the adverse phase exhibited marked divergence (*p* = 0.001), coinciding with the upregulation of inflammation-associated metabolic pathways (as detailed below). In contrast, the establishment of retinol tolerance induced partial metabolic regression toward baseline (adverse → tolerance: *p* = 0.001), while retaining adaptive adjustments (baseline → tolerance: *p* = 0.003). These findings suggest that a new functional equilibrium was gradually achieved through the dynamic modulation of metabolic processes.

MetOrigin analysis (Figure 3C) provided further insight. Firstly, a comparison between the baseline and the time point at which an adverse reaction occurred revealed a marked upregulation of several lipid and fatty acid metabolic pathways in the skin. Notably, ko00564 (Glycerophospholipid metabolism), ko00591 (Linoleic acid metabolism), ko00592 (alpha-Linolenic acid metabolism), and ko00565 (Ether lipid metabolism) exhibited significant increases in activity. This upregulation suggests that early exposure to exogenous retinol triggers these metabolic pathways, resulting in membrane lipid remodeling. The activation of these pathways, particularly those involved in arachidonic acid and linoleic acid metabolism, are typically associated with inflammatory mediator production, which may ultimately compromise local barrier function. Additionally, the activation of ko00260 (Glycine, serine, and threonine metabolism) and ko00680 (Methane metabolism) implies that cells and associated microbial communities under stress elevate their demand for amino acid metabolism and energy regulation. Furthermore, a comparison between the adverse reaction phase and the tolerance established phase indicated that several additional pathways were significantly upregulated in the skin. Specifically, enhanced activities were observed in ko00600 (Sphingolipid metabolism), ko00020 (Citrate cycle, i.e., TCA cycle), hsa00053 (Ascorbate and aldarate metabolism), and hsa00040 (Pentose and glucuronate interconversions). This pattern suggests that following the initial phase of inflammation and barrier disruption, the skin engages in reparative processes by reestablishing energy metabolism, enhancing antioxidant defenses and detoxification capabilities, and repairing the barrier. Concurrently, the downregulation of pathways associated with inflammation and lipid mediator production–including ko00564, ko00591, ko00592, ko00565, and ko00590 (Arachidonic acid metabolism)—indicates a progressive suppression of inflammatory signals that paves the way for retinol tolerance.

Notably, the significant fluctuations in metabolomic α-diversity stand in sharp contrast to the relative stability of microbial α-diversity observed in Section 3.2, positioning metabolites as more sensitive biomarkers for monitoring cutaneous adaptation to retinol. The dramatic metabolomic fluctuations contrasted with the relative microbial stability, suggesting that metabolites serve as more sensitive indicators of skin adaptation. To identify predictive biomarkers, we next compared baseline characteristics between tolerant and intolerant groups.

### 3.4 Multi-omics profiling of baseline characteristics between retinol-tolerant and -intolerant groups

**Figure 4:**
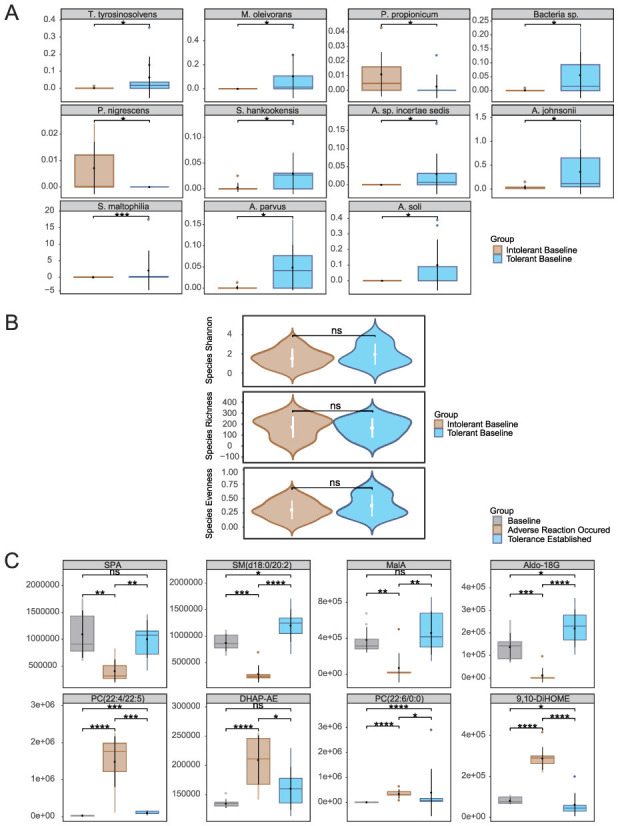
Baseline multi-omics profiling of retinol-tolerant vs. intolerant groups: **(A)** Differentially siginificant species, **(B)** α-diversity metrics, and **(C)** Quantitative comparison of key metabolites. Species abbreviations: *T. tyrosinosolvens, Tsukamurella tyrosinosolvens*; *M. oleivorans, Microbacterium oleivorans*; *P. propionicum, Pseudopropionibacterium propionicum*; *Bacteria sp., Bacteria* species; *A. parvus, Acinetobacter parvus*; *P. nigrescens, Prevotella nigrescens*; *S. hankookensis, Sphingomonas hankookensis*; *A. sp. incertae sedis, Acidovorax sp. incertae sedis*; *A. johnsonii, Acinetobacter johnsonii*; *A. soli, Acinetobacter soli*; *S. maltophilia, Stenotrophomonas maltophilia*. Metabolites abbreviations: DHAP-AE, Dihydroxyacetone Phosphate Acyl Ester; 9,10-DiHOME, (9s,10s) 9,10Dihydroxyoctadecanoic Acid; SPA, Sphinganine; MalA, Malic Acid; Aldo-18G, Aldosterone 18Glucuronid). *p < 0.05, **p < 0.01, ***p < 0.001, ****p < 0.0001.

Integrated analysis of baseline microbiome and metabolome data (Day 0) unveiled distinct signatures differentiating the retinol-tolerant and retinol-intolerant groups. Microbiome analysis revealed that the intolerant group exhibited a higher baseline abundance of *Cutibacterium acnes*, suggesting that its pre-treatment overgrowth may contribute to increased retinol sensitivity. In contrast, the tolerant group exhibited greater diversity among low-abundance species, indicative of a more balanced ecological community. Baseline species abundance comparisons using Wilcoxon tests, followed by Benjamini–Hochberg correction, identified 11 species with statistically significant differential abundance (Figure 4A). Taxa such as *Sphingomonas hankookensis* and *Acinetobacter johnsonii* were enriched in the tolerant group, potentially facilitating retinol tolerance, whereas the intolerant group was characterized by a higher prevalence of species like *Prevotella nigrescens*, which may promote inflammation. In agreement with previous studies (Larsen, 2017), Prevotella species have been implicated in driving mucosal Th17-mediated inflammation. Notably, our earlier analysis found that within the intolerant group, the abundance of *P. nigrescens* increased during the development of intolerance and subsequently decreased once tolerance was achieved, further supporting its role in eliciting inflammatory responses. Despite these compositional distinctions, overall microbial α-diversity metrics showed no significant differences between the groups (Figure 4B), implying that variations in retinol tolerance are driven by the functional regulation of key taxa rather than by wholesale restructuring of the microbial community. KEGG enrichment analysis (Supplementary Figure 3) revealed divergent metabolic strategies: the intolerant group upregulated pathways related to flavonoid biosynthesis (map00941) and plant hormone signaling (map04075), both associated with antioxidant defense, whereas the tolerant group activated pathways involved in autophagy (map04136) and endoplasmic reticulum protein processing (map04141), indicative of enhanced proteostasis.

Metabolomic analysis (Figure 4C) identified eight metabolites that not only exhibited significant baseline differences between the tolerant and intolerant groups but were also implicated in previously enriched metabolic pathways likely influencing retinol tolerance. Their dynamic expression patterns revealed group-specific responses with distinct temporal trajectories. In the intolerant group, these metabolites demonstrated two contrasting kinetic trends: one subset initially declined in response to retinol stimulation before rebounding, whereas another subset was upregulated early and then gradually waned as tolerance became established. In contrast, the tolerant group (Supplementary Figure 4) exhibited a markedly different metabolic profile, with only four of the eight metabolites being detectable (the remaining four were below the instrumental detection limit), and among these four detectable metabolites, significant temporal changes were observed across the three time points. These differential kinetics underscore the coordinated interplay of multiple metabolic regulatory networks during the skin's stress response and subsequent repair processes.

In the intolerant group, malic acid from the TCA cycle (ko00020) and aldosterone 18-glucuronide—detected within the Pentose and glucuronate interconversions as well as Ascorbate and aldarate metabolism pathways—exhibited an initial decrease followed by a recovery. The early decline likely reflects a transient disruption in energy metabolism and a temporary compromise of antioxidant defenses under retinol-induced stress, while the subsequent rebound suggests a gradual restoration of metabolic homeostasis, reestablishing energy supply and activating detoxification mechanisms essential for skin repair. Notably, in the tolerant group, malic acid remained stable throughout the treatment period with no significant changes, suggesting preserved energy metabolism and metabolic resilience, while aldosterone 18-glucuronide showed a significant increase from day 0 to day 21, potentially indicating enhanced constitutive detoxification capacity without the initial metabolic disruption observed in intolerant individuals. Similarly, sphinganine and SM(d18:0/20:2) from the sphingolipid metabolism pathway (ko00600) displayed analogous dynamics in the intolerant group; their early reduction may indicate stress-induced consumption or remodeling of membrane lipids, and their recovery is likely instrumental in restoring membrane integrity and normalizing cellular signaling. However, the tolerant group demonstrated a distinctly different sphingolipid response pattern: sphinganine decreased significantly from day 0 to day 7, while SM(d18:0/20:2) showed a significant decline from day 0 to day 21. This sustained reduction in sphingolipid levels, contrasting with the recovery pattern seen in intolerant individuals, may reflect more efficient membrane adaptation mechanisms that preemptively facilitate tolerance without requiring metabolic recovery phases. Additionally, phospholipid metabolites associated with membrane regulation and inflammatory mediator generation—including various phosphatidylcholine (PC) isomers, Dihydroxyacetone Phosphate Acyl Ester, and (9s,10s)-9,10-Dihydroxyoctadecanoic Acid—were upregulated during the initial response in the intolerant group, reflecting disturbances in membrane composition and the activation of inflammatory cascades. As retinol tolerance developed, these metabolites gradually declined, suggesting an effective suppression of inflammatory signaling and a steady progression of barrier repair. Importantly, these inflammatory-associated phospholipid metabolites were undetectable in the tolerant group, suggesting that tolerance may be fundamentally characterized by the absence of inflammatory metabolic activation rather than its subsequent resolution. To understand how these microbial and metabolic changes interact to drive tolerance establishment, we performed integrated multi-omics correlation analysis.

### 3.5 Functional integration of multi-omics correlation networks

**Figure 5:**
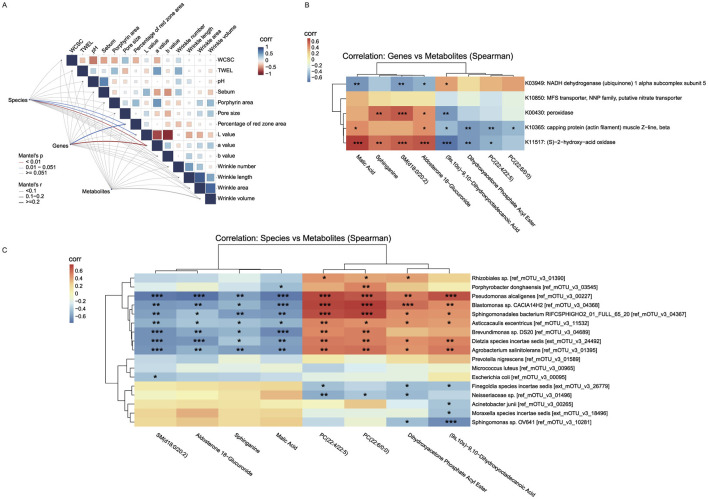
Correlation backbone of the unified working model of retinol tolerance. **(A)** Mantel correlation matrix linking multi-omics distance matrices with skin phenotypes (line thickness indicates correlation strength; colors denote significance). **(B)** Gene–metabolite correlation heatmap (KEGG Orthology vs. key metabolites), which we use as the correlation-based unified working model aligning microbial functional signals with metabolite classes across phases. **(C)** Species–metabolite correlation heatmap highlighting mirror associations between transiently changing taxa and lipid/energy-related metabolites. All patterns represent associations; causality is not inferred. Mantel matrix visualization: Line thickness indicates correlation strength (thin: |*r*| < 0.1, mostly negative; thick: 0.1 ≤ *r* ≤ 0.2, positive); colors denote significance (red: *p* < 0.01; blue: 0.01 < *p* < 0.051; gray: non-significant). “Corr” labels reflect Pearson autocorrelations of skin phenotypes (blue: positive, red: negative). *p < 0.05, **p < 0.01, ***p < 0.001.

Mantel analysis (Figure 5A) detected weak but statistically significant correlations between microbial abundance and erythema-related phenotypes. Specifically, the percentage of red zone area (*r* = 0.164, *p* = 0.044), skin lightness (L*) (*r* = 0.168, *p* = 0.042), and the red-green a* value (*r* = 0.184, *p* = 0.005) all showed weak but statistically significant correlations with microbial abundance. Furthermore, the abundance of microbial genes was significantly correlated with the value of a * (*r* = 0.336, *p* = 0.002) and the percent of the area of the red zone (*r* = 0.250, *p* = 0.033). Furthermore, the metabolomic profile did not show any direct correlations with phenotypic traits (*p*>0.05), suggesting that its role may be mediated through more complex interactions.

Using a gene–metabolite correlation heatmap (Figure 5B) based on the eight metabolites that showed significant changes in the intolerant group (as described in Figure 4C) and five genes that exhibited sustained significant changes in the intolerant group, we observed close interactions between key genes and metabolites. Specifically, genes K00430, K10365, and K11517 showed significant positive correlations with Sphinganine, SM(d18:0/20:2), Malic acid, and Aldosterone 18-glucuronide (with correlation coefficients of approximately +0.35 to + 0.73), and significant negative correlations with phosphatidylcholine (PC) metabolites, Dihydroxyacetone Phosphate Acyl Ester, and (9s,10s)-9,10-Dihydroxyoctadecanoic Acid (with coefficients of approximately −0.17 to −0.72). This “mirror” correlation effect suggests that under retinol-induced stress, the partial recovery of cellular energy metabolism and antioxidant defenses (evidenced by a rebound in metabolites linked to the TCA cycle and sphingolipid metabolism) is accompanied by the upregulation of these genes. Such regulation likely contributes to balancing energy supply and redox homeostasis while mitigating the accumulation of PC metabolites that results from membrane damage or inflammatory activation. In contrast, K03949 displayed a negative correlation with metabolites associated with the TCA cycle and sphingolipid metabolism (correlation coefficients approximately −0.38 to −0.58) and a positive correlation with PC metabolites (correlation coefficients approximately +0.17 to +0.39). This pattern suggests that, during the early phase of retinol-induced stress, K03949 may engage in compensatory regulation of energy metabolism in response to mitochondrial dysfunction or energy deficits, thereby promoting membrane lipid repair.

Similarly, using a species–metabolite correlation heatmap (Figure 5C) that integrated the same eight significantly altered metabolites from the intolerant group with the 17 microbial species that showed significant changes in the intolerant group (as described in Figure 2B), we observed that many species which exhibited significant changes following skin stress (e.g., *Pseudomonas alcaligenes, Blastomonas sp., Agrobacterium salinitolerans, Brevundimonas sp., Asticcacaulis excentricus*, and *Dietzia species incertae sedis*) were strongly, and statistically significantly, negatively correlated with Sphinganine, SM(d18:0/20:2), Malic acid, and Aldosterone 18-glucuronide (correlation coefficients ranging approximately from −0.4 to −0.76). Conversely, these species were significantly positively correlated with PC metabolites [e.g., PC(22:4/22:5) and PC(22:6/0:0)], Dihydroxyacetone Phosphate Acyl Ester, and (9s,10s)-9,10-Dihydroxyoctadecanoic Acid (with correlation coefficients typically above 0.5 and extremely significant *p*-values). This mirror correlation indicates that, during the early stage of retinol-induced stress, increases in the abundance of certain microbial taxa are closely linked to fluctuations in metabolites associated with cellular energy metabolism, antioxidant defense, and membrane lipid remodeling. Specifically, the suppressed TCA cycle and sphingolipid metabolism lead to reduced levels of Malic acid, Sphinganine, and SM, while disturbances in the cell membrane are reflected by the accumulation of PC metabolites. Overall, this inverse correlation pattern further supports the notion of an imbalanced local skin environment during the initial phase of retinol stimulation. At this early stage, cellular protective mechanisms (such as energy replenishment and antioxidant capacity) are transiently compromised, while phospholipid metabolites involved in membrane repair and signal transduction are rapidly upregulated to counteract local inflammation and barrier disruption. As skin tolerance gradually develops, both the microbial abundances and the levels of these metabolites return to homeostasis, revealing the close interconnection between microbial and host metabolic regulation.

## 4 Discussion

This study offers a comprehensive, multi-omics perspective on biological processes associated with skin responses to topical retinol. Our analyses are consistent with a phase-specific interplay between host metabolic pathways and the resident microbial community during cutaneous remodeling. We observed patterns compatible with two phases: an acute adaptation phase, marked by a pronounced decline in specific metabolite classes and rapid shifts in microbial composition, and a re-equilibration phase during which key energy and lipid metabolic pathways are reactivated to facilitate the restoration of barrier function.

During the early acute response (baseline → adverse reaction), *in vivo* metabolomic data were consistent with a relative reduction in the TCA cycle and sphingolipid metabolism, accompanied by increased phospholipid remodeling and an upregulation of fatty acid oxidation pathways. These metabolic alterations may reflect an early shift toward signaling lipids and inflammatory mediators. The transient surge in membrane-derived phosphatidylcholines (PCs) is consistent with established mechanisms of membrane perturbation and phospholipase activation under stress conditions (Zhong et al., 2019), underscoring the role of membrane remodeling in the onset of retinol intolerance. Concurrently, transient increases in taxa such as *Agrobacterium salinitolerans, Blastomonas sp*., and *Pseudomonas alcaligenes* further suggest that opportunistic microbes can proliferate when the barrier is compromised (Byrd et al., 2018).

In addition to taxa associated with resilient skin ecologies in tolerant individuals (e.g., *Sphingomonas hankookensis, Acinetobacter johnsonii*), our data also highlighted organisms with pathogenic/pathobiont potential. At baseline, the intolerant group exhibited higher abundance of *Cutibacterium acnes* and a greater prevalence of *Prevotella nigrescens*, consistent with, but not diagnostic of, a community state more permissive to irritant responses. During the acute adverse-reaction phase in the intolerant group, several environmental/opportunistic taxa—predominantly Gram–negative (including *Pseudomonas alcaligenes, Brevundimonas* sp., *Agrobacterium salinitolerans*, and *Blastomonas* sp.), as well as *Dietzia* (Gram–positive)—displayed a transient “rise–and–fall” pattern. Species–metabolite correlations linked these increases to higher phosphatidylcholines and linoleic-acid–derived oxylipins (e.g., 9,10-DiHOME), together with lower levels of TCA–cycle and sphingolipid intermediates, a profile consistent with membrane remodeling and pro–inflammatory lipid signaling under barrier stress. Notably, we did not observe a cohort-wide, consistent expansion of canonical skin pathogens such as *Staphylococcus aureus*. In contrast, the tolerant group showed comparatively stable community structure with fewer transient fluctuations (limited shifts were observed for *P. nigrescens* and *Sphingomonas* sp.), suggesting that baseline ecological composition and barrier context may constrain pathobiont proliferation upon retinol challenge. Taken together, these patterns support a model in which barrier perturbation and opportunistic microbial shifts co-occur with inflammatory lipid dynamics; the temporal ordering remains unresolved.

During the transition from the adverse reaction phase to tolerance establishment, increased TCA-cycle intermediates and recovery of sphingolipid precursors (e.g., sphinganine) coincided with declines in phosphatidylcholines and oxylipins, suggesting partial metabolic re-equilibration. Enrichment of ascorbate/aldarate metabolism and pentose/glucuronate interconversions points to enhanced redox buffering and detoxification (Bickers and Athar, 2006). These associations are time-aligned but non-causal. In parallel, transient microbial shifts attenuated and functional repertoires stabilized, supporting a co-adaptive trajectory between host metabolism and the microbiome. Viewed together, this time-ordered pattern is operationalized by the gene-metabolite heatmap (Figure 5B), which serves as a unified “working model” aligning KO-level functional signals with metabolite classes across the two phases. The opposing (mirror) alignment of K00430/K10365/K11517 vs. K03949 with recovery/repair vs. remodeling/inflammation markers exemplifies the transition from early intolerance to tolerance. We stress that this representation is associative; establishing directionality will require targeted perturbations and flux-based validation.

Baseline multi-omics comparisons between retinol-tolerant and retinol-intolerant individuals underscore the potential predictive value of specific microbial and metabolic biomarkers. Tolerant subjects exhibited higher baseline proportions of *Sphingomonas hankookensis* and *Acinetobacter johnsonii*—taxa that have been reported to be stress-resilient; in our data, higher baseline abundance of these taxa was associated with tolerance. Whether they facilitate barrier integrity or modulate inflammatory responses requires testing (Cosseau et al., 2016; Seifert et al., 1997). Moreover, distinctive baseline lipid precursors in the tolerant group, such as malic acid and sphingolipid intermediates, are compatible with greater capacity to sustain energy homeostasis and maintain membrane stability under stress; definitive functional inference would require flux-based assays. These observations may inform translational studies that tailor retinol regimens based on an individual's baseline microbial and metabolic profiles.

Nonetheless, several limitations of this study warrant acknowledgment. First, despite rigorous statistical controls (paired designs, permutation-based PERMANOVA/Adonis, and BH–FDR), our sample size is small (*n* = 18), which limits power for high-dimensional inference and generalizability. Accordingly, findings should be validated in larger, independent cohorts with longitudinal sampling in both groups. Second, the metabolic origin of specific metabolites—particularly those that may derive from both host and microbial sources—requires validation through methods such as stable-isotope tracing to elucidate metabolic fluxes. Third, a more comprehensive lipidomic analysis is needed to better discriminate the roles of different lipid classes (e.g., ceramides v. phosphatidylcholines) in orchestrating barrier dynamics in response to retinol-induced stress. Finally, while our data capture longitudinal shifts in barrier parameters and multi-omics profiles, further mechanistic studies, including *in vitro* or *ex vivo* models, are warranted to dissect the interactions between individual microbial taxa and host epithelial cells under retinol challenge. Moreover, the observational, single-arm design and absence of a vehicle-only control preclude causal attribution and raise the possibility of unmeasured confounding.

To address generalizability, we plan a prospective external validation in an independent, larger cohort focused on a parsimonious baseline panel: a microbial log-ratio of *Cutibacterium acnes* to *Sphingomonas*/*Acinetobacter*, baseline sebum and skin pH, and targeted metabolites (malic acid, sphinganine). A brief validation protocol is outlined in Supplementary methods.

These observations may inform translational studies for personalized retinoid care. The identification of baseline biomarkers—particularly elevated *Sphingomonas hankookensis* and specific sphingolipid intermediates in tolerant individuals—if prospectively validated, could help stratify patients by retinol tolerance risk. Such stratification could motivate evaluation of prophylactic barrier-support strategies in clinical trials (e.g., ceramide supplementation or microbiome-oriented optimization) prior to retinol initiation. In addition, barrier- and microbiome-targeted adjuncts may be explored: ceramide- and linoleic-acid–rich moisturizers and niacinamide for barrier priming; antioxidant co-care with topical ascorbate derivatives (consistent with the enrichment of ascorbate/aldarate metabolism during tolerance); and cautious prebiotic/postbiotic approaches intended to discourage *Cutibacterium acnes* overgrowth while supporting commensals (e.g., sphingoid-base–containing formulations). Furthermore, the dynamic metabolic signatures we identified (TCA cycle intermediates, sphinganine levels) could serve as candidate monitoring biomarkers pending prospective validation, enabling precision-guided dose escalation and treatment optimization beyond traditional subjective symptom assessment. A “start–low, go–slow” initiation algorithm (e.g., alternate–day use, a moisturizer-retinol-moisturizer “sandwich,” and predefined down–titration or temporary holds upon early intolerance markers) could be explored in pragmatic pilot studies; clinical adoption should await evidence from controlled trials. Objective phenotypes (WCSC, TEWL, pH, sebum) could serve as exploratory monitoring endpoints; in research settings, periodic swab/tape–strip metabolomics may be evaluated as candidate pharmacodynamic markers.

Building upon these findings, several important research priorities emerge for advancing precision dermatology. Larger validation cohorts encompassing diverse ethnic populations and age groups would be valuable to confirm the generalizability of our predictive biomarker signatures. Interventional studies testing targeted co-treatments—such as topical ceramide formulations, probiotic or postbiotic approaches involving beneficial taxa (*Sphingomonas, Acinetobacter*), or metabolic modulators targeting sphingolipid synthesis—merit investigation to determine their potential in preventing retinol intolerance. Extending this multi-omics framework to other retinoids (tretinoin, adapalene, tazarotene) and cosmeceutical actives (glycolic acid, vitamin C) could reveal shared tolerance-associated mechanisms and broaden the clinical utility of predictive profiling. Moreover, mechanistic validation through controlled *in vitro* co-culture systems and ex vivo skin models would be important for dissecting specific host–microbe interactions and testing targeted interventions. Longitudinal studies with extended follow-up periods may help optimize treatment protocols by identifying optimal re-introduction timing and maintenance strategies for sustained tolerance. It is worth noting that live 'next-generation' topical probiotics (e.g., Sphingomonas/Acinetobacter) remain investigational and will require rigorous safety testing and regulatory evaluation before clinical adoption.

In summary, our findings are consistent with a tightly coupled host–microbe response to retinol exposure, featuring an initial metabolic perturbation followed by patterns compatible with reconstitution of cutaneous homeostasis. The transient decoupling, followed by the reconvergence of key metabolic pathways—namely TCA cycle activity, sphingolipid biosynthesis, and phospholipid signaling—highlights patterns compatible with retinol tolerance. Collectively, these observations provide a foundation for future precision dermatology interventions, which, if validated, could inform the development of pre- or co-treatment strategies aimed at bolstering sphingolipid metabolism and barrier function in individuals at risk of retinol intolerance.

## Data Availability

The datasets and analysis code used in this study are available on GitHub: https://github.com/Yxhuang1024/HBN_project.

## Footnotes

^1^ https://metorigin.met-bioinformatics.cn/home/
