# Supplementary figures and images for: Dynamic multi-omics mechanisms underpinning retinol tolerance: stage-specific reconstruction of skin barrier function and host–microbiome metabolic interactions
Source: Front Microbiol. 2025 Oct 27;16:1668712. doi: 10.3389/fmicb.2025.1668712 (PMC12598784; doi:10.3389/fmicb.2025.1668712)

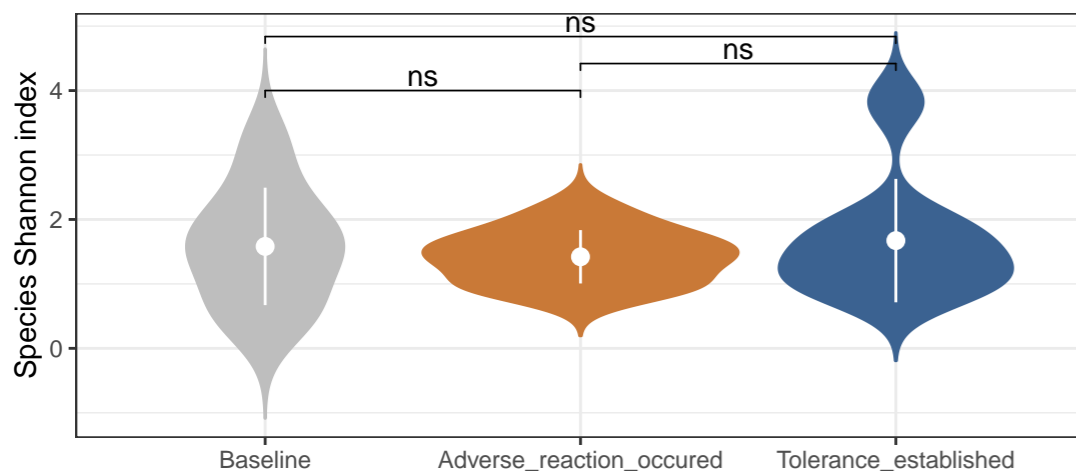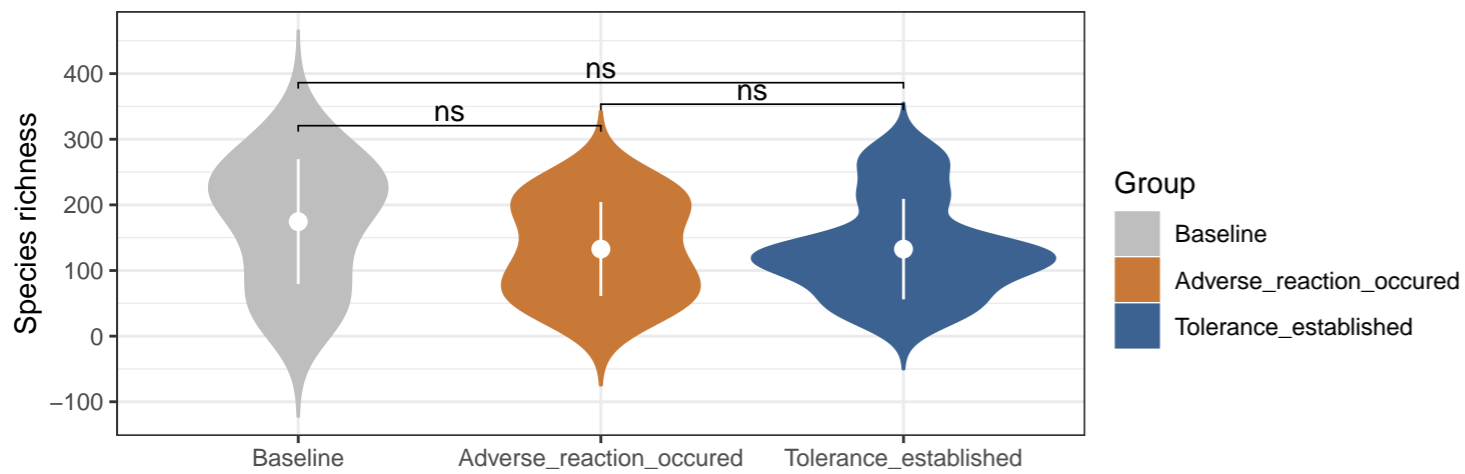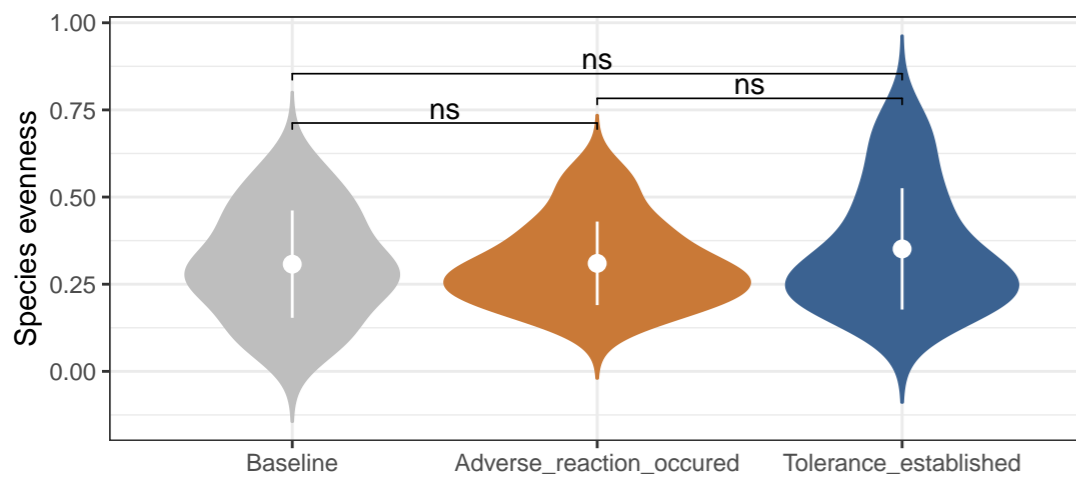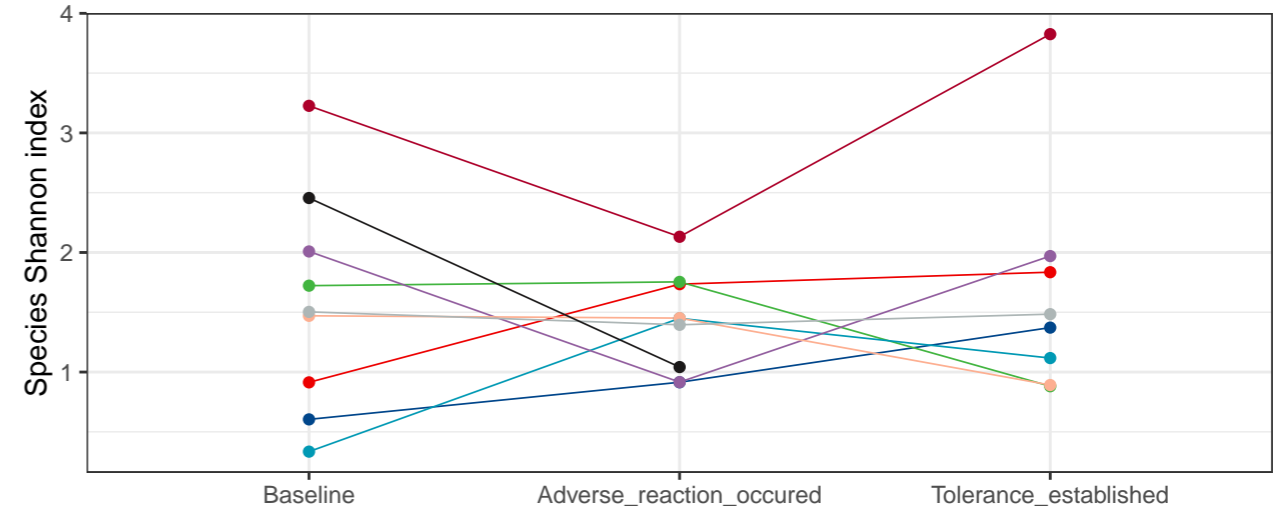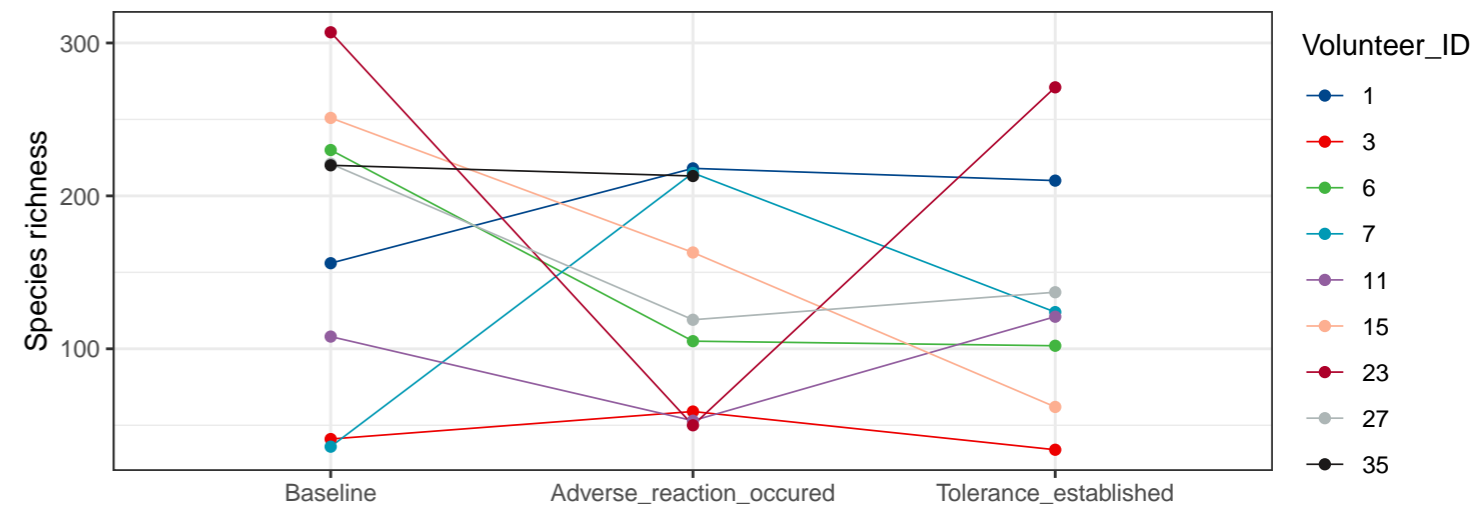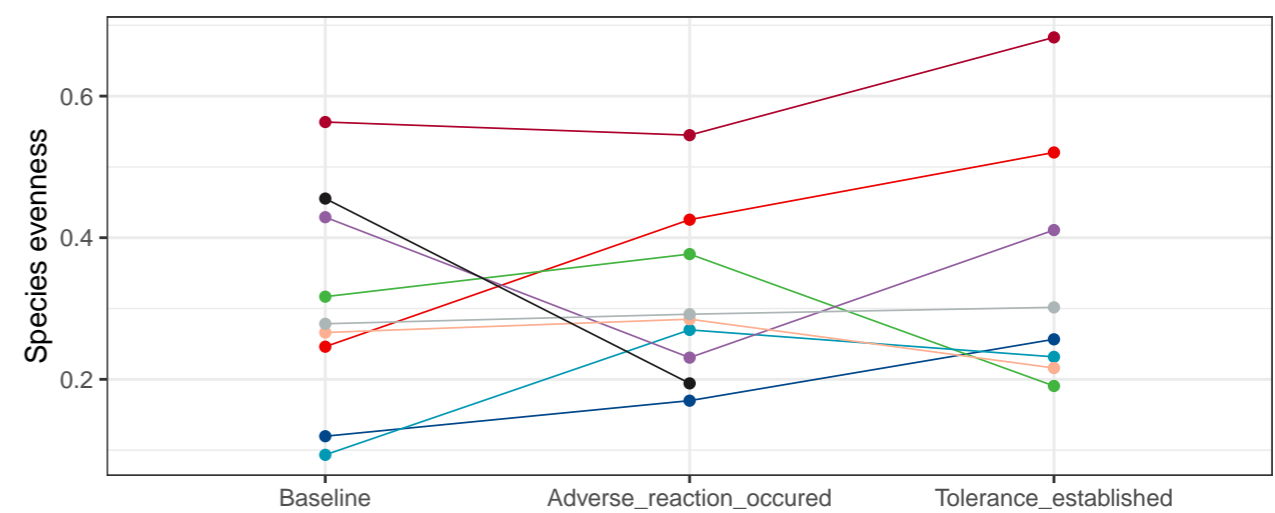

Supplement: Supplementary file 1 [file Image_1.pdf]

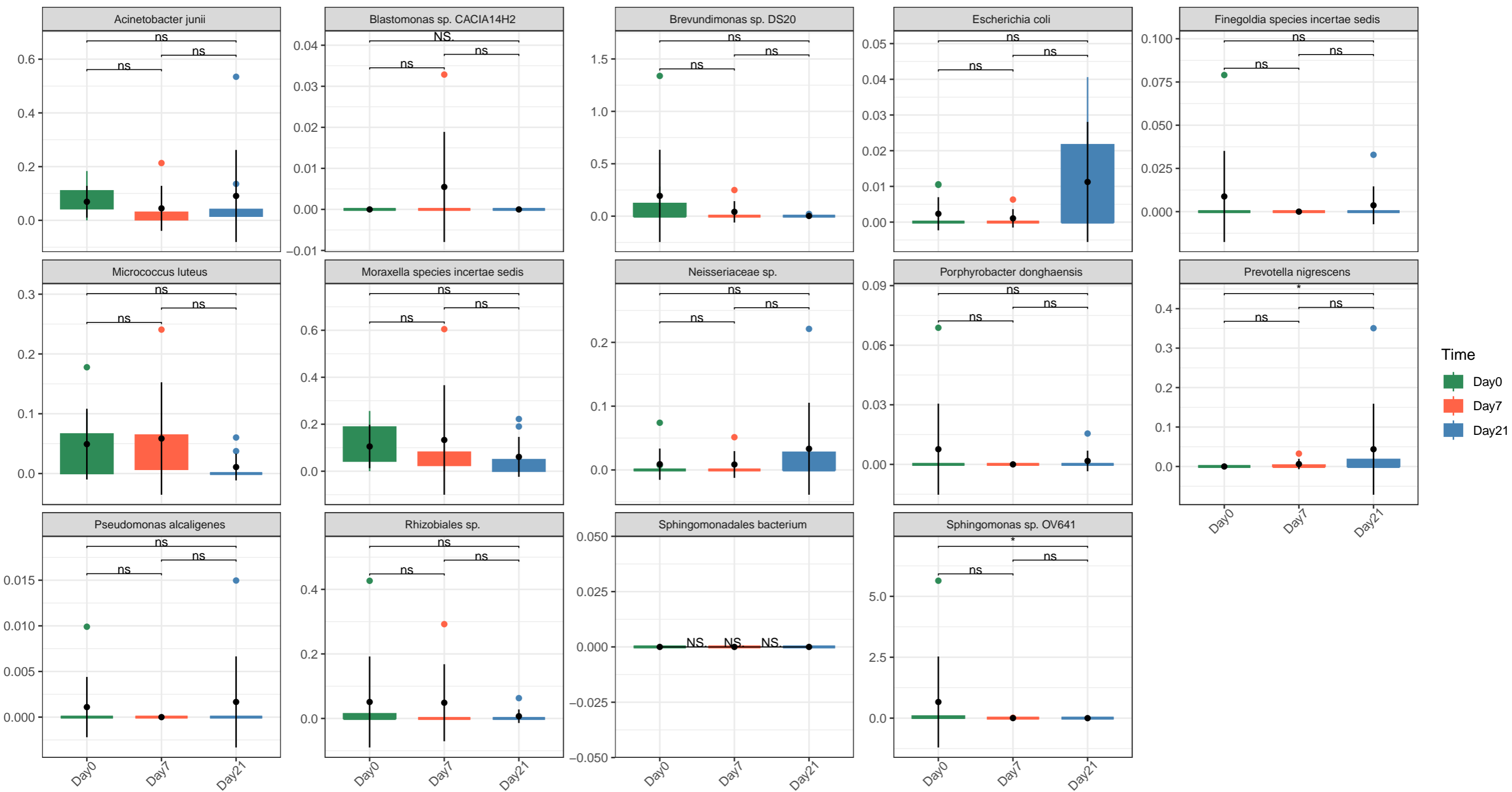

Supplement: Supplementary file 2 [file Image_2.pdf]

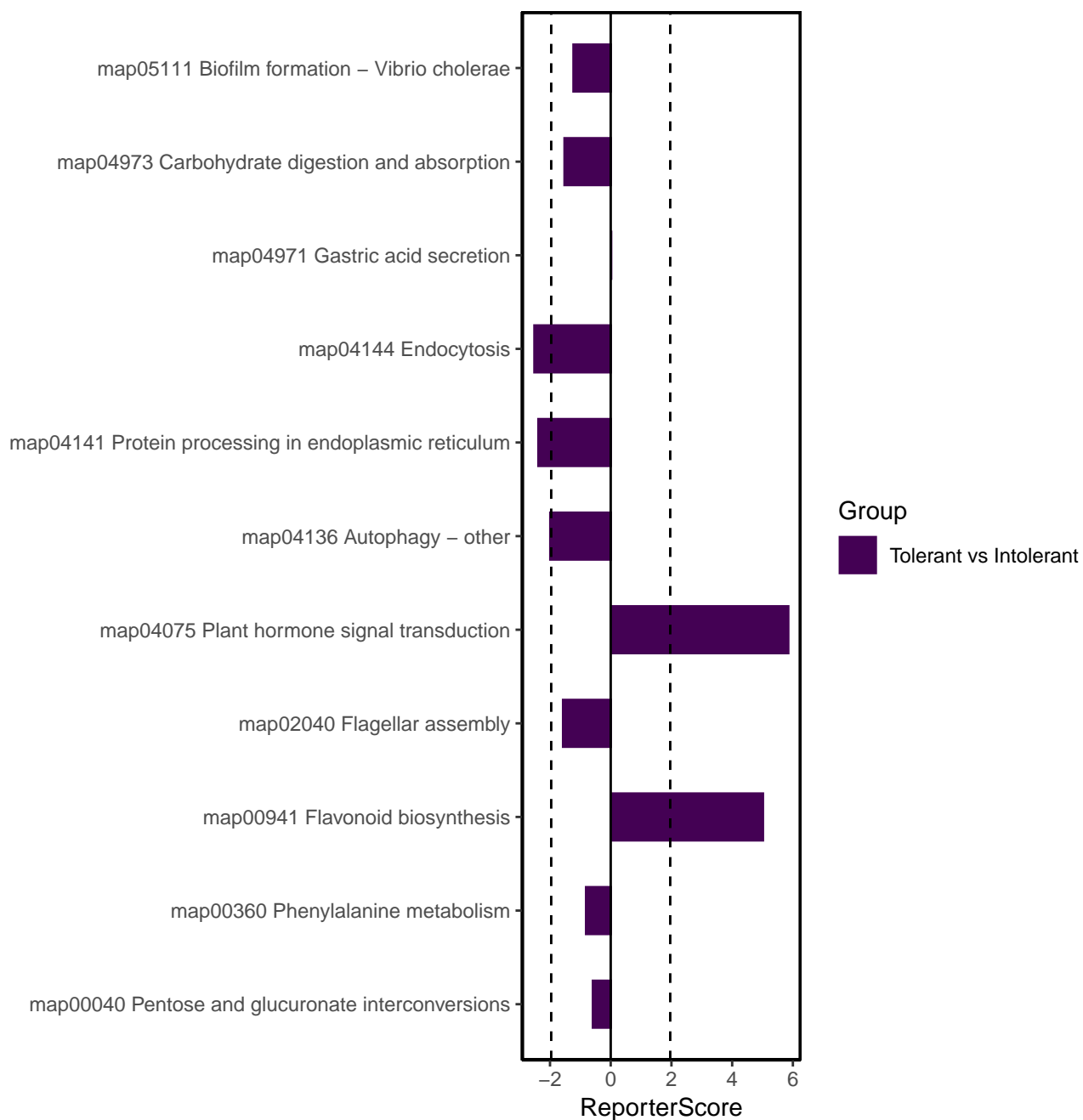

Supplement: Supplementary file 3 [file Image_3.pdf]

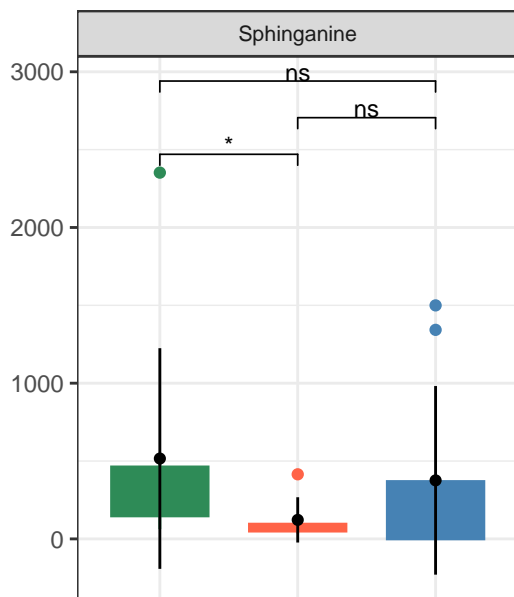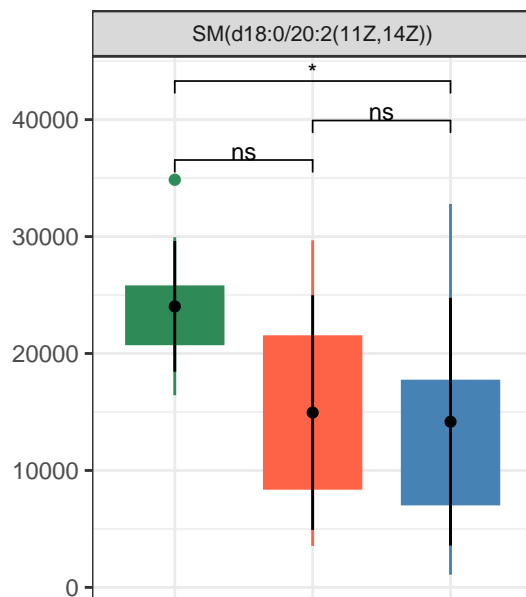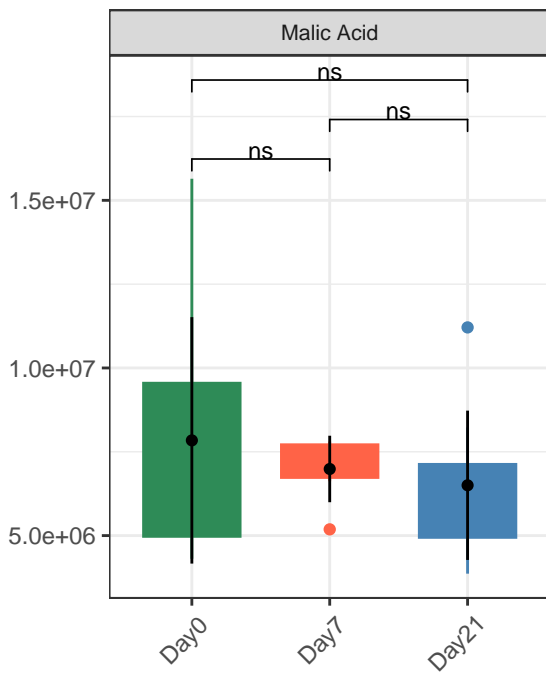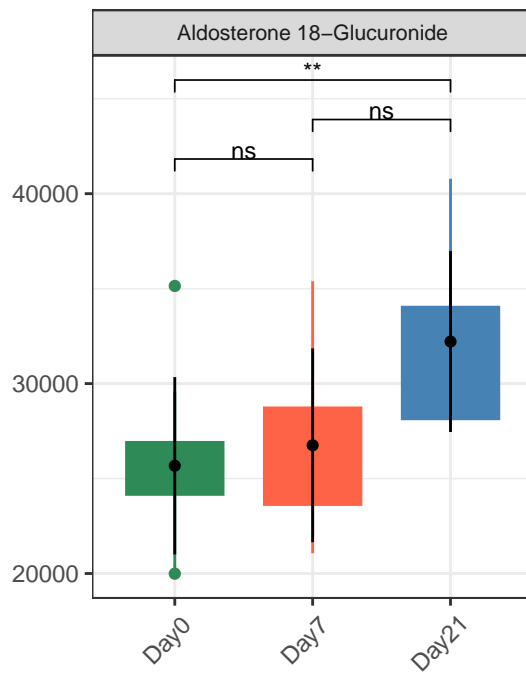

Time

- Day0
- Day7
- Day21

Supplement: Supplementary file 4 [file Image_4.pdf]
